# In Vitro Activity of 3-Bromopyruvate, an Anticancer Compound, Against Antibiotic-Susceptible and Antibiotic-Resistant *Helicobacter pylori* Strains

**DOI:** 10.3390/cancers11020229

**Published:** 2019-02-15

**Authors:** Paweł Krzyżek, Roman Franiczek, Barbara Krzyżanowska, Łukasz Łaczmański, Paweł Migdał, Grażyna Gościniak

**Affiliations:** 1 Department of Microbiology, Faculty of Medicine, Wroclaw Medical University, Wroclaw 50-368, Poland; roman.franiczek@umed.wroc.pl (R.F.); barbara.krzyzanowska@umed.wroc.pl (B.K.); grazyna.gosciniak@umed.wroc.pl (G.G.); 2 Hirszfeld Institute of Immunology and Experimental Therapy, Polish Academy of Sciences, Wroclaw 53-114, Poland; lukasz@diagmol.com; 3 Department of Environment, Hygiene and Animal Welfare, Wroclaw University of Environmental and Life Sciences, Wroclaw 51-630, Poland; pawel.migdal@upwr.edu.pl

**Keywords:** coccoid forms, checkerboard assay, gastric cancer, time-killing assay

## Abstract

*Helicobacter pylori (H. pylori)* is a bacterium capable of inducing chronic active gastritis, which in some people, develops into gastric cancers. One of the substances that may be useful in the eradication of this microorganism is 3-Bromopyruvate (3-BP), an anticancer compound with antimicrobial properties. The aim of this article was to determine the activity of 3-BP against antibiotic-susceptible and antibiotic-resistant *H. pylori* strains. The antimicrobial activity was determined using a disk-diffusion method, broth microdilution method, time-killing assay, and checkerboard assay. The research was extended by observations using light, fluorescence, and scanning electron microscopy. The growth inhibition zones produced by 2 mg/disk with 3-BP counted for 16–32.5 mm. The minimal inhibitory concentrations (MICs) ranged from 32 to 128 μg/mL, while the minimal bactericidal concentrations (MBCs) for all tested strains had values of 128 μg/mL. The time-killing assay demonstrated the concentration-dependent and time-dependent bactericidal activity of 3-BP. The decrease in culturability below the detection threshold (<100 CFU/mL) was demonstrated after 6 h, 4 h, and 2 h of incubation for MIC, 2× MIC, and 4× MIC, respectively. Bacteria treated with 3-BP had a several times reduced mean green/red fluorescence ratio compared to the control samples, suggesting bactericidal activity, which was independent from an induction of coccoid forms. The checkerboard assay showed the existence of a synergistic/additive interaction of 3-BP with amoxicillin, tetracycline, and clarithromycin. Based on the presented results, it is suggested that 3-BP may be an interesting anti-*H. pylori* compound.

## 1. Introduction

*Helicobacter pylori* is a Gram-negative, flagellated, spiral-shaped rod inhabiting the human gastric mucosa [1]. It has been estimated that nearly 4.4 billion (over 60%) of people in the world are colonized with this bacterium, of which the highest prevalence was recorded in Africa (70.1%) and the lowest in Oceania (24.4%) [2]. This pathogen spreads from person to person, leading to the persistent stomach colonization and the development of chronic active gastritis [3]. As a result of this colonization, lasting for many decades, some people may develop a sequence of histopathological gastric changes promoting the formation of tumors [4,5]. Due to the ability of *H. pylori* to induce carcinogenesis, this bacterium was classified in 1994 by the International Agency for Research on Cancer as a group I carcinogen [6].

Gastric cancers are the fifth most common cancers and the third most frequent cause of cancer-dependent deaths in the world [7,8]. The development of gastric cancers is a complex, multistep process that leads to a series of genetic and epigenetic changes within signaling factors, cell cycle regulators, and tumor suppressor genes. Despite its multifactorial nature, it is estimated that in about 80% of cases, *H. pylori* is responsible for the formation of gastric cancers [9]. Therefore, the eradication of this bacterium before the appearance of significant and irreversible changes in the gastric mucosa may protect against the development of gastric cancer [10,11,12].

One of the main challenges in anti-*H. pylori* therapies is the growing resistance of this bacterium to antibiotics [3]. The level of antibiotic resistance has reached alarming levels around the world. Primary and secondary resistance to clarithromycin (CLR), metronidazole (MTZ), and levofloxacin exceeded the value of ≥15% in virtually all areas within the World Health Organization (WHO) framework, which currently makes them unable to be use in empirical therapies. Double, secondary resistance to both CLR and MTZ also reached a worrying level, exceeding 10% in the Eastern Mediterranean region, the Western Pacific region, and Europe, with the highest recorded prevalence in Europe (18%) [13]. Due to the increasing antibiotic resistance of many microorganisms around the world, in 2017, WHO published a list of highest priority bacteria that need searching for new antimicrobial substances, among which CLR-resistant *H. pylori* was mentioned [14]. One of such substances that may be useful in the future in the eradication of drug-resistant *H. pylori* is 3-Bromopyruvate (3-BP).

3-BP is a chemically synthesized halogen pyruvic acid analogue [15,16]. High interest in this substance is associated with anti-oncogenic activity directed against various types of cancer cells [16]. The first report on the anticancer properties of 3-BP was published in 2001 [17], which contributed to the appearance of many scientific reports indicating the selective action of 3-BP against various cancer cells [16], including in vitro [18] and in vivo [19] studies targeting gastric cancer. The cytotoxic activity against cancer cells is associated with the promotion of metabolic catastrophe, leading to interference with the activity of glycolytic enzymes and mitochondrial respiration proteins, the limitation of intracellular ATP and the induction of oxidative stress [15,16]. Additionally, antimicrobial activity of 3-BP directed against protozoa [20,21], fungi [22,23], microalgae [24], and bacteria [25,26] has also been demonstrated.

Due to the numerous beneficial therapeutic properties of 3-BP, the aim of this article was to determine the activity of this compound against antibiotic-susceptible and antibiotic-resistant *H. pylori* strains, both alone and in combination with the most commonly used antibiotics. The study was also extended to assess the effect of 3-BP on the *H. pylori* morphology.

## 2. Results

### 2.1. Disk-Diffusion Method

The first stage of research was a screening test of the 3-BP activity against *H. pylori* strains using the disk-diffusion method. The sizes of growth inhibition zones were concentration-dependent and counted for 16–32.5 mm, 10–28.5 mm, and 6–19 mm for 2000 μg/disk, 1000 μg/disk, and 200 μg/disk, respectively (Table 1). The antibiotic resistance profile of tested *H. pylori* strains had no significant effect on the size of the growth inhibition zones (*p* > 0.05, Tables S1–S4). Despite the high activity of 3-BP at the highest concentration used (zones of inhibition ˃ 15 mm), these values were much lower than those obtained for amoxicillin (AMX), which were in the range of 58.5–70.5 mm.

### 2.2. Determination of MICs and MBCs

To determine the minimal inhibitory concentration (MIC) and minimal bactericidal concentration (MBC) values of 3-BP, ten *H. pylori* strains with different antibiotic resistance profiles were selected (Table 2). The lowest MICs were observed in antibiotic-susceptible clinical *H. pylori* strains and corresponded to concentrations of 32–64 μg/mL. The highest MICs were recorded against the reference and double-resistant clinical strains (128 μg/mL). For MTZ-resistant and CLR-resistant strains, the MICs were 32–128 μg/mL and 64–128 μg/mL, respectively. For all *H. pylori* strains tested, the MBCs counted for 128 μg/mL, whereas the MBC/MIC ratios were ≤4, indicating a bactericidal 3-BP activity against *H. pylori* strains (Table 2). Besides, the differences in the 3-BP activity between *H. pylori* strains were independent of the antibiotic resistance profile (*p* > 0.05). Moreover, it was shown that the activity of 3-BP against two reference *H. pylori* strains (Tx30a and J99) was pH-independent and that both MICs and MBCs had values of 128 μg/mL.

### 2.3. Time-Killing Assay

**Figure 1:**
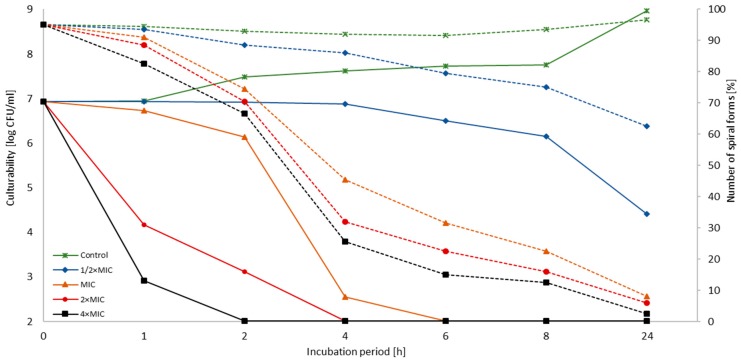
The effect of 3-Bromopyruvate (3-BP) on the culturability and morphology of *H. pylori* Tx30a during the incubation period. The number of spiral forms is marked with dotted lines, while the culturability is indicated by continuous lines.

**Figure 2:**
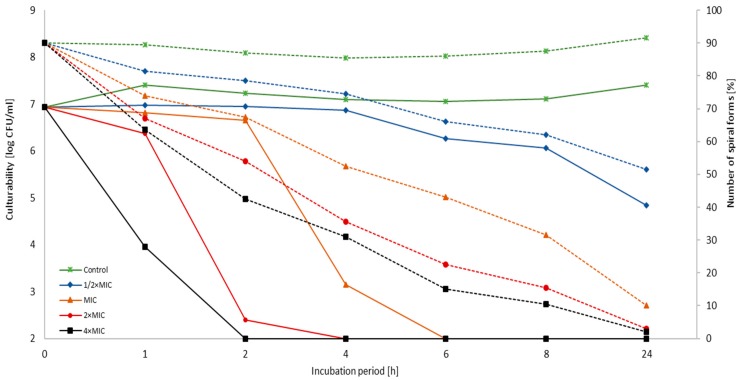
The effect of 3-Bromopyruvate (3-BP) on the culturability and morphology of *H. pylori* J99 during the incubation period. The number of spiral forms is marked with dotted lines, while the culturability is indicated by continuous lines.

The next step of the study was designated to determine the morphology, culturability, and viability of the two reference *H. pylori* strains (Tx30a and J99) during the incubation with 3-BP. There were no statistically significant differences in the tested parameters between these strains (*p* > 0.05). For both, the concentration-dependent and time-dependent activity of 3-BP was demonstrated (Figure 1 and Figure 2).

For both strains, a decrease in the culturability below the detection threshold (˂100 CFU/mL) was demonstrated after 6 h, 4 h, and 2 h for MIC, 2× MIC, and 4× MIC of 3-BP, respectively (Figure 1 and Figure 2). The steep decline in bacterial counts at 2× MIC and 4× MIC has been observed. For MIC, there was a linear, slow decrease in the first 2 h incubation (reduction in log_10_ CFU/mL < 1), with a dynamic reduction in the bacterial counts between 2 h and 4 h, i.e., from 10^6.13^ to 10^2.54^ (*H. pylori* Tx30a) and from 10^6.65^ to 10^3.14^ (*H. pylori* J99) (Tables S5 and S6). The dynamics of the decrease in the culturability of tested strains treated with MIC-4× MIC of 3-BP were significantly different in comparison to both the control (Tx30a: *p* < 0.0005 at all concentrations; J99: *p* < 0.0005 for 2× MIC and 4× MIC and *p* < 0.005 for MIC) and ½× MIC (Tx30a and J99: *p* < 0.005 for 2× MIC and 4× MIC and *p* < 0.05 for MIC).

**Figure 3:**
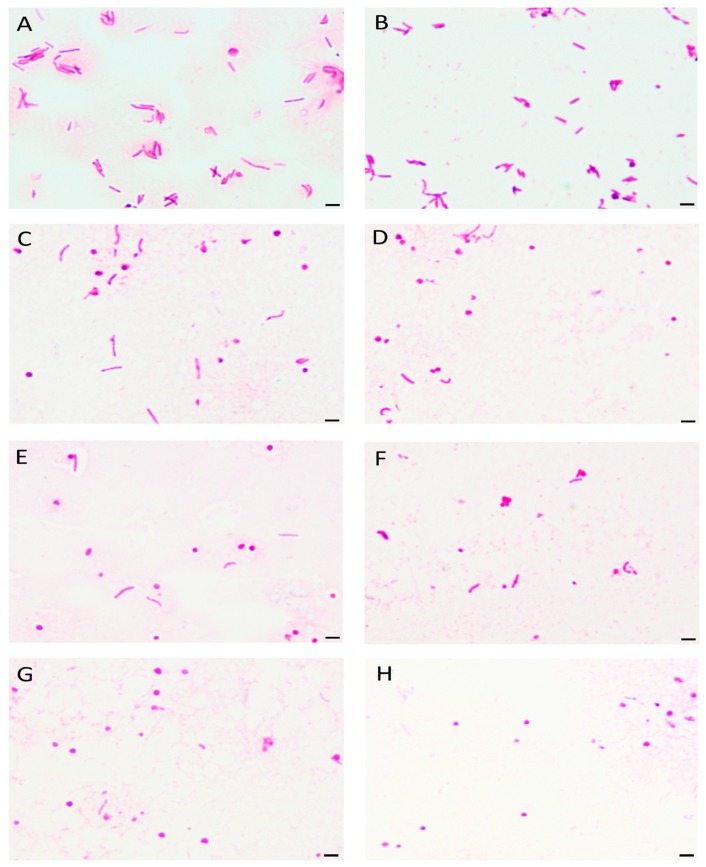
The light microscopy of *H. pylori* cells during the incubation with 3-Bromopyruvate (3-BP): The representative microscopic images of *H. pylori* cells during the incubation with the MIC of 3-BP after (**A**) 0 h, (**B**) 1 h, (**C**) 2 h, (**D**) 4 h, (**E**) 6 h, (**F**) 8 h, and (**G**) 24 h show a time-dependent decrease in the number of spiral forms with an inversely proportional increase in the number of coccoid forms. The negative control consisted of bacterial cells (**H**) after a 1 h treatment with 70% ethanol. The scale bar in the light microscopy is 2 μm.

**Figure 4:**
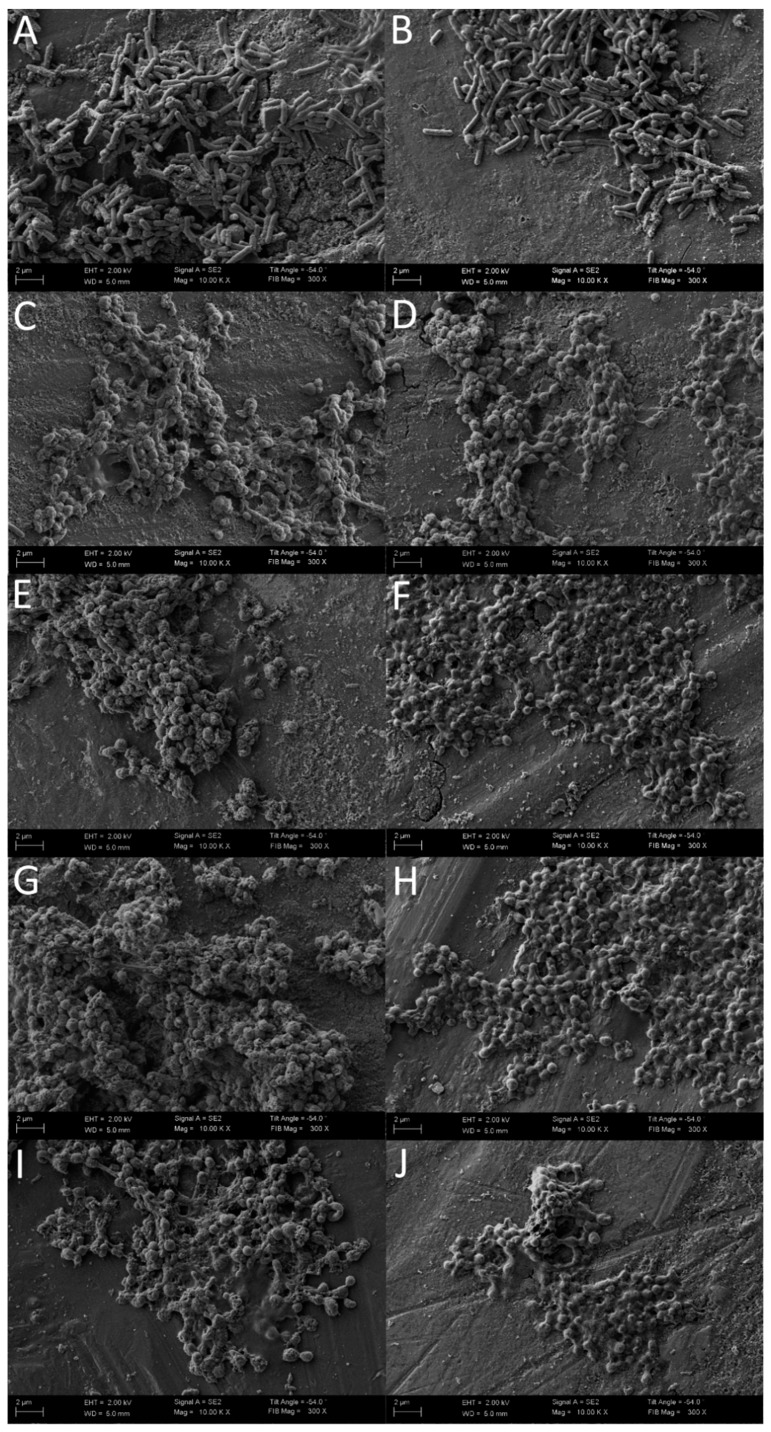
The scanning electron microscopy images of *H. pylori* Tx30a (**A**,**C**,**E**,**G**,**I**) and J99 (**B**,**D**,**F**,**H**,**J**) cells (**A**,**B**) without 3-Bromopyruvate (3-BP), seen mainly as spiral forms, and treated with different concentrations of 3-BP: (**C**,**D**) ½× MIC, (**E**,**F**) MIC, (**G**,**H**) 2× MIC, and (**I**,**J**) 4× MIC, in which the coccoid forms predominate.

The reduction of bacterial culturability was accompanied by a decrease in the number of spiral forms with an inversely proportional increase in the number of coccoid forms (Figure 1, Figure 2 and Figure 3). No differences were observed in the dynamics of the decrease in spiral forms between the tested *H. pylori* strains at any time point (*p* > 0.05). For both strains, a linear decrease in the number of spiral forms over time was observed for each 3-BP concentration tested. Differences in the number of spiral forms between the control and samples treated with 3-BP were statistically significant in a 1 h incubation (*p* < 0.05) and highly significant for all time points starting from the 2 h incubation (*p* < 0.0000). Both *H. pylori* strains after the one-day cultivation period occurred mainly in the spiral form (>90%). This morphotype was practically unobservable for bacteria treated with MIC-4× MIC of 3-BP. For the Tx30a strain, the average number of cells with the spiral morphology in a 24 h incubation counted for 2.5%, 6%, and 8% for 4× MIC, 2× MIC, and MIC, respectively (Figure 1 and Table S7), whereas for J99 strain, the average amount of these morphological forms was 2%, 3%, and 10%, respectively (Figure 2 and Table S8). The morphological variability of *H. pylori* strains exposed to 3-BP was also documented by a scanning electron microscopy. At a 72 h incubation, in MIC-4× MIC treated samples, both strains were practically exclusively spherical (Figure 4C–J). However, differences in the amount of exopolysaccharide, an important component of biofilm, were noticed. The exopolysaccharide matrix was present in a great amount in the case of *H. pylori* J99 treated with 3-BP and practically absent during the observation of *H. pylori* Tx30a. In the control samples not treated with 3-BP, both strains occurred mainly in the spiral form (Figure 4A,B).

**Figure 5:**
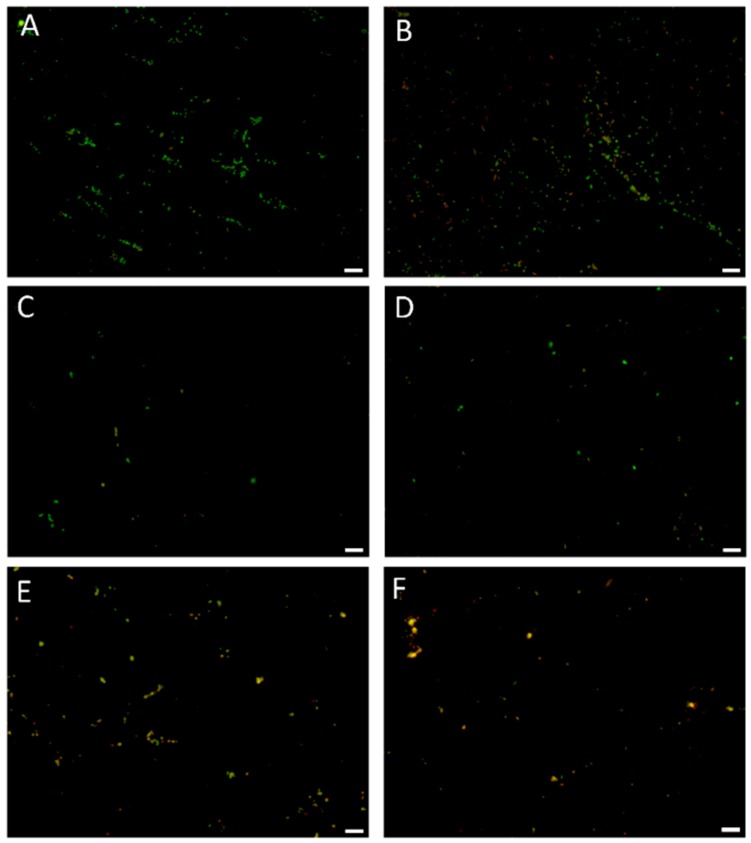

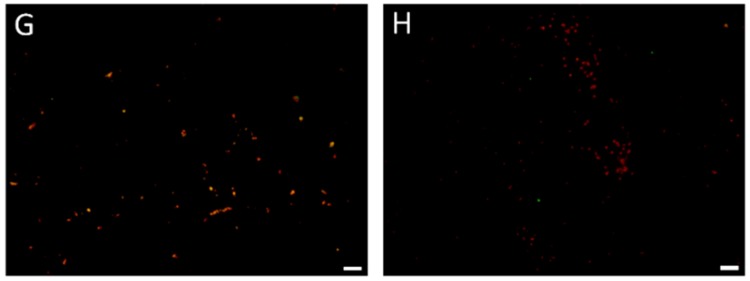
The fluorescence microscopy of *H. pylori* cells during the incubation with 3-Bromopyruvate (3-BP): The representative microscopic images of *H. pylori* cells during the incubation with the MIC of 3-BP after (**A**) 0 h, (**B**) 1 h, (**C**) 2 h, (**D**) 4 h, (**E**) 6 h, (**F**) 8 h, and (**G**) 24 h. The negative control consisted of bacterial cells (**H**) after a 1 h treatment with 70% ethanol. The green cells indicate live bacteria, whereas the red/orange cells indicate damaged, dead bacteria. The scale bar in the fluorescence microscopy is 20 μm.

**Figure 6:**
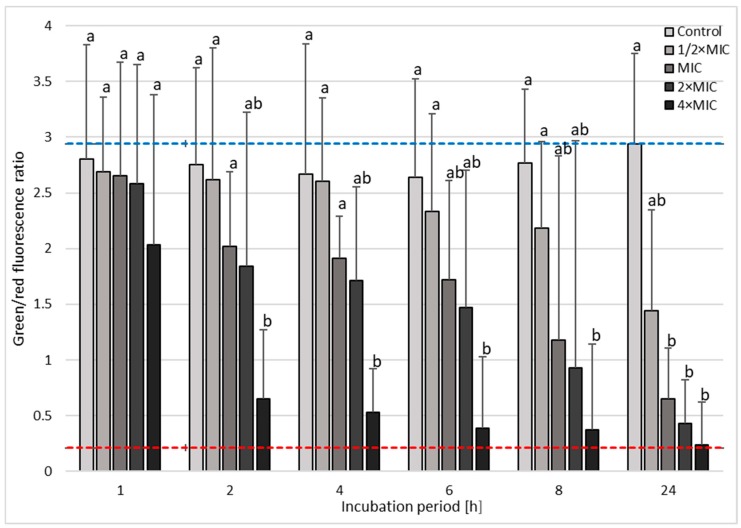
The fluorescence microscopy analysis of the viability during the incubation of *H. pylori* Tx30a with 3-Bromopyruvate (3-BP) in time. The blue top and red bottom lines indicate the positive (0 h incubation) and negative controls (1 h treatment with 70% ethanol), respectively. Columns with the same subscript letters are not significantly different from each other (*p* > 0.05).

**Figure 7:**
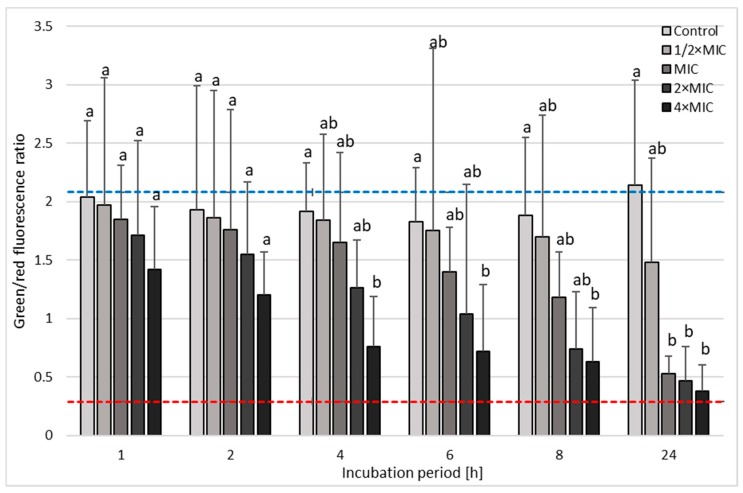
The fluorescence microscopy analysis of the viability during the incubation of *H. pylori* J99 with 3-Bromopyruvate (3-BP) in time. The blue top and red bottom lines indicate the positive (0 h incubation) and negative controls (1 h treatment with 70% ethanol), respectively. Columns with the same subscript letters are not significantly different from each other (*p* > 0.05).

The confirmation of bactericidal, concentration-dependent, and time-dependent activity of 3-BP against *H. pylori* strains was performed using fluorescence microscopy (Figure 5). In the case of *H. pylori* Tx30a, statistically significant differences were found in the mean green/red fluorescence ratio between tested samples after 1 h incubation (*p* < 0.05), with an observable increase in statistical significance for all time points starting from 2 h of incubation (*p* < 0.0000). After a one-day incubation period, the mean green/red fluorescence ratio for the control sample was 12-fold, 7-fold, and 4.5-fold higher than in the samples treated with 4× MIC, 2× MIC, and MIC of 3-BP, respectively (Figure 6 and Table S9). A similar tendency was observed for *H. pylori* J99, although in this case, differences between the tested samples were highly significant starting from 1 h after incubation (*p* < 0.0000). After 24 h of incubation, the mean green/red fluorescence ratio for this strain in the control sample was over 4–5 times higher than in MIC-4× MIC of 3-BP (Figure 7 and Table S10). The analysis of the differences in the mean green/red fluorescence ratio within a given concentration of 3-BP at various time points showed a statistically significant decrease in green fluorescence intensity over time for all tested 3-BP concentrations for *H. pylori* Tx30a (*p* < 0.0000) and MIC-4× MIC for *H. pylori* J99 (*p* < 0.0000). For the *H. pylori* J99, there were no significant changes in the green/red fluorescence intensity during the incubation of this strain with ½× MIC of 3-BP (*p* > 0.05).

### 2.4. Checkerboard Assay

**Figure 8:**
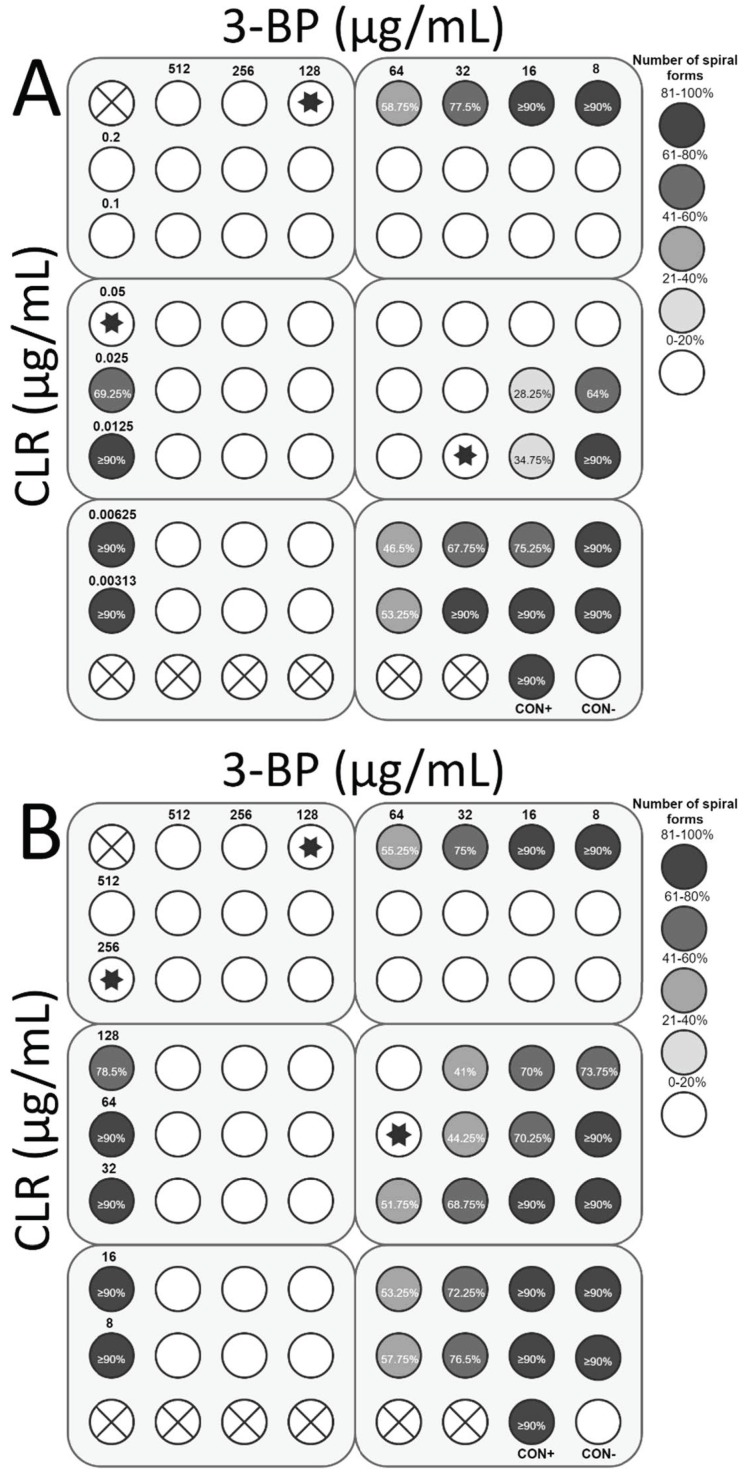
The antibacterial and morphological effects of 3-Bromopyruvate (3-BP), clarithromycin (CLR), and combinations of both against *H. pylori* Tx30a and 7143 strains: The existence of the interaction in the antimicrobial activity of 3-BP with CLR was determined against (**A**) the reference antibiotic-susceptible *H. pylori* Tx30a and (**B**) the clinical double-resistant *H. pylori* 7143 strain. The white circles indicate the wells in which the number of spiral forms was ≤15%, while the white circles with a cross in the middle indicate empty wells. Using asterisks, the wells with the MICs of the tested substances were marked, whereas in the case of the interaction verifications, they indicate the lowest FIC. Abbreviations: 3-BP, 3-Bromopyruvate; CLR, Clarithromycin; CON+, Positive control; and CON−, Negative control.

**Figure 9:**
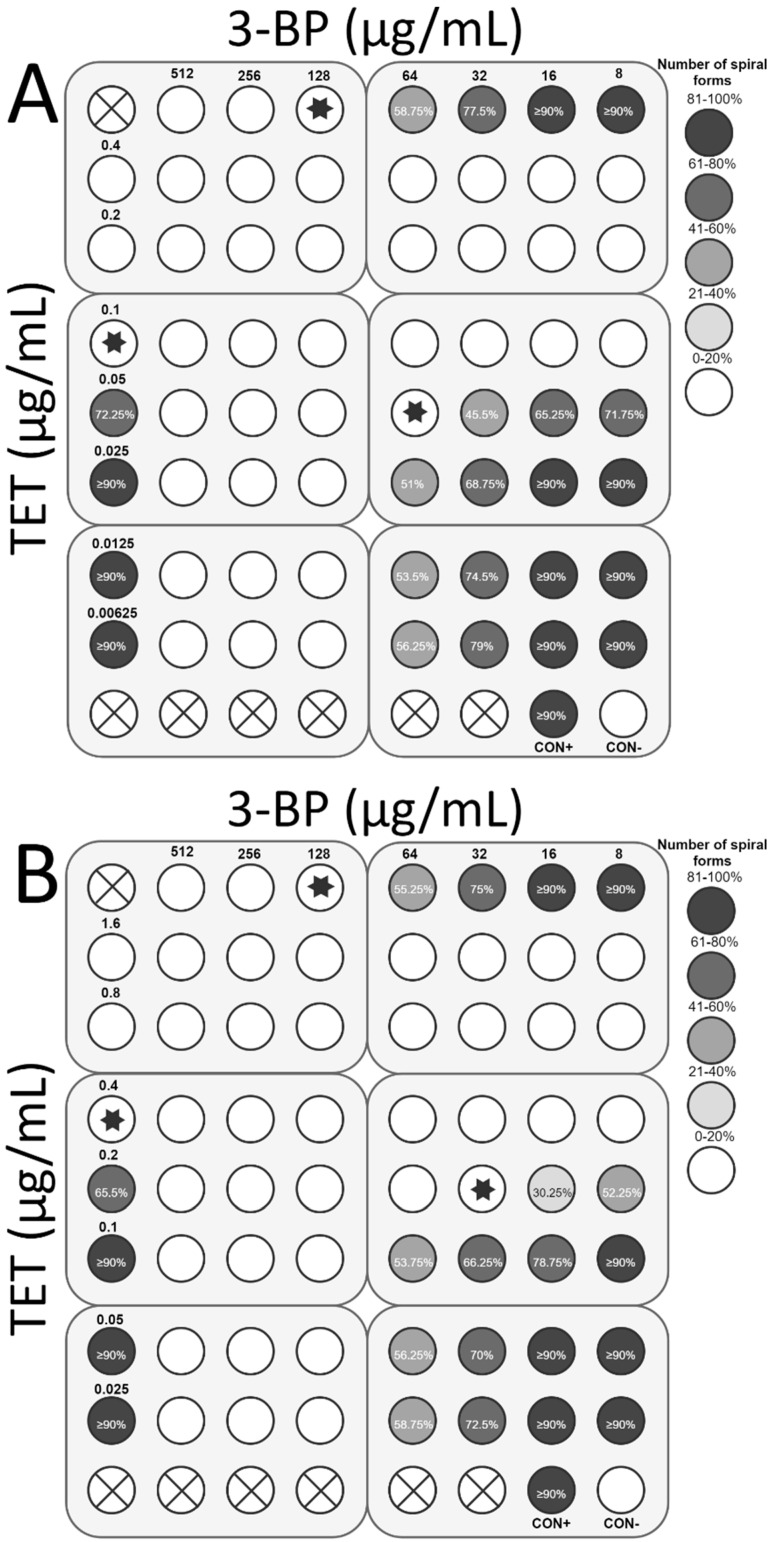
The antibacterial and morphological effects of 3-Bromopyruvate (3-BP), tetracycline (TET), and combinations of both against *H. pylori* Tx30a and 7143 strains. The existence of the interaction in the antimicrobial activity of 3-BP with CLR was determined against (**A**) the reference antibiotic-susceptible *H. pylori* Tx30a and (**B**) the clinical double-resistant *H. pylori* 7143 strain. The white circles indicate the wells in which the number of spiral forms was ≤15%, while the white circles with a cross in the middle indicate empty wells. Using asterisks, the wells with the MICs of the tested substances were marked, whereas in the case of the interaction verifications, they indicate the lowest FIC. Abbreviations: 3-BP, 3-Bromopyruvate; TET, Tetracycline; CON+, Positive control; and CON−, Negative control.

**Figure 10:**
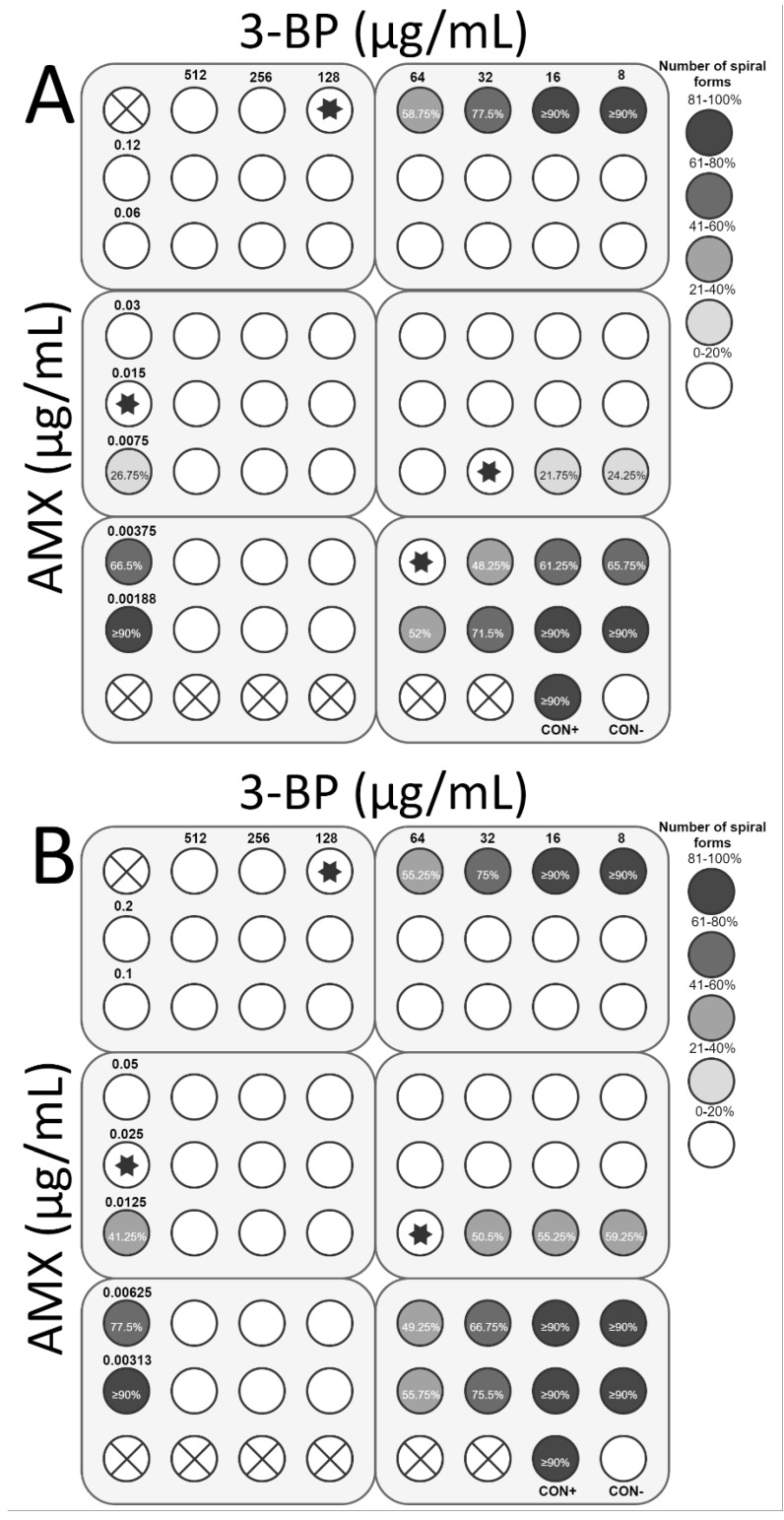
The antibacterial and morphological effects of 3-Bromopyruvate (3-BP), amoxicillin (AMX), and combinations of both against *H. pylori* Tx30a and 7143 strains. The existence of the interaction in the antimicrobial activity of 3-BP with AMX was determined against (**A**) the reference antibiotic-susceptible *H. pylori* Tx30a and (**B**) the clinical double-resistant *H. pylori* 7143 strain. The white circles indicate the wells in which the number of spiral forms was ≤15%, while the white circles with a cross in the middle indicate empty wells. Using asterisks, the wells with the MICs of the tested substances were marked, whereas in the case of the interaction verifications, they indicate the lowest FIC. Abbreviations: 3-BP, 3-Bromopyruvate; AMX, Amoxicillin; CON+, Positive control; and CON−, Negative control.

**Figure 11:**
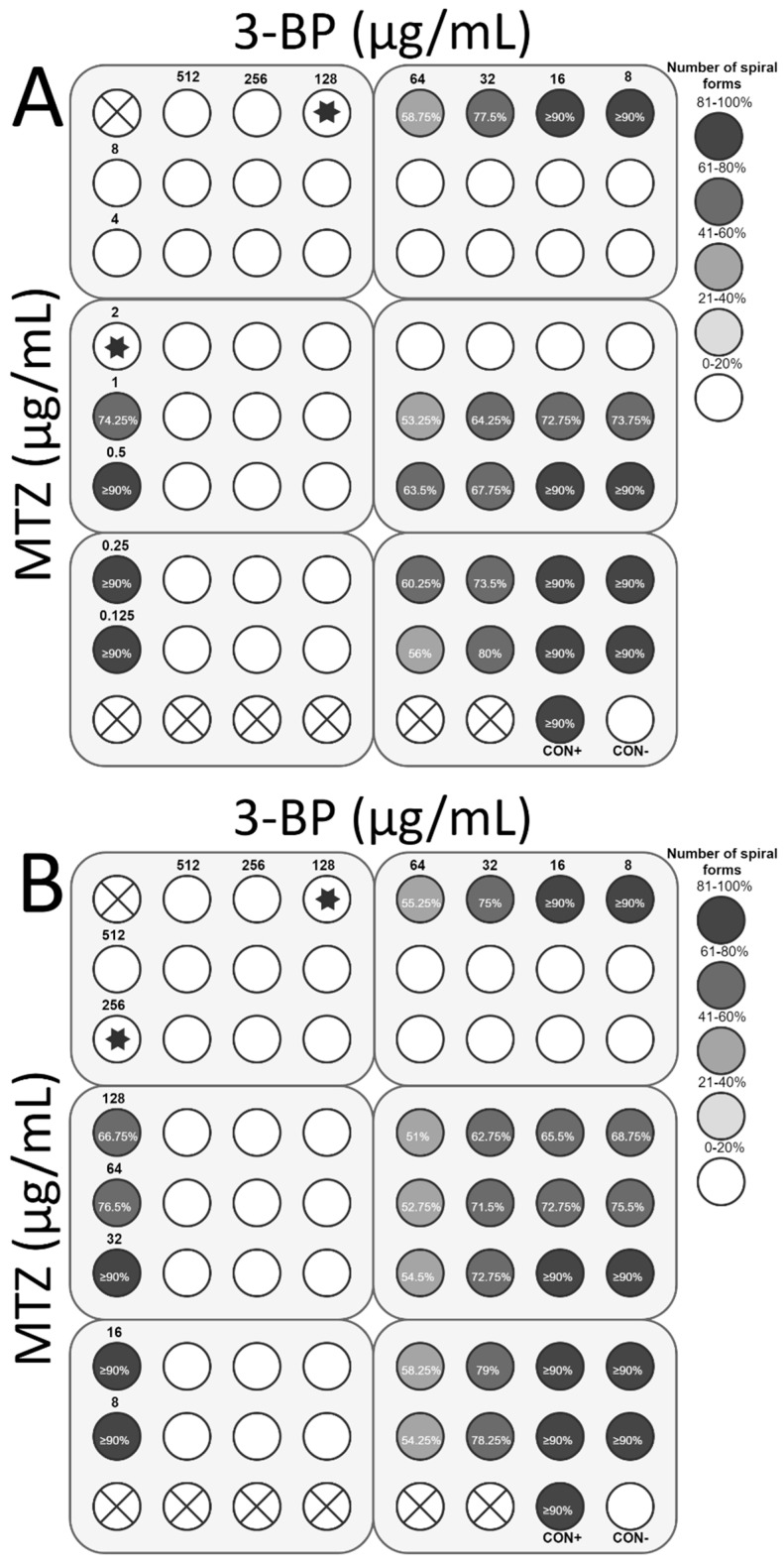
The antibacterial and morphological effects of 3-Bromopyruvate (3-BP), metronidazole (MTZ), and combinations of both against *H. pylori* Tx30a and 7143 strains. The existence of the interaction in the antimicrobial activity of 3-BP with MTZ was determined against (**A**) the reference antibiotic-susceptible *H. pylori* Tx30a and (**B**) the clinical double-resistant *H. pylori* 7143 strain. The white circles indicate the wells in which the number of spiral forms was ≤15%, while the white circles with a cross in the middle indicate empty wells. Using asterisks, the wells with the MICs of the tested substances were marked, whereas in the case of the interaction verifications, they indicate the lowest FIC. Abbreviations: 3-BP, 3-Bromopyruvate; MTZ, Metronidazole; CON+, Positive control; and CON−, Negative control.

The next stage of the study was to determine the interaction of 3-BP with the most commonly used antibiotics in *H. pylori* therapy, i.e., AMX, TET, CLR, and MTZ. For *H. pylori* Tx30a, the synergistic activity of 3-BP with CLR (FIC = 0.5) was demonstrated, which was accompanied by a 4-fold reduction in concentration of both substances (from 128 μg/mL to 32 μg/mL and from 0.05 μg/mL to 0.0125 μg/mL, respectively) while maintaining antimicrobial activity against the tested strain (Figure 8A). In addition, an additive interaction of 3-BP with AMX (FIC = 0.75) and TET (FIC = 1) was found (Figure 9A and Figure 10A). On the other hand, no interaction between 3-BP and MTZ was observed (FIC = 2) (Figure 11A). Similar results were obtained for the *H. pylori* 7143 strain. For AMX (FIC = 1) and TET (FIC = 0.75), the presence of additive interaction with 3-BP was shown (Figure 9B and Figure 10B). The combination of CLR and 3-BP also indicated an additive interaction (FIC = 0.75), whereas the presence of 3-BP, similar to the Tx30a strain, resulted in a 4-fold decrease in the MIC of CLR (from 256 μg/mL to 64 μg/mL) (Figure 8B). Besides, for the *H. pylori* 7143 strain, no interaction between 3-BP and MTZ was observed (FIC = 2) (Figure 11B).

The checkerboard assay was additionally expanded to analyze the morphology of *H. pylori* treated with 3-BP and the four antibiotics mentioned above (Table S11–S18). It was found that in the MICs of all tested substances, *H. pylori* underwent a morphological transformation into a coccoid form (˃85% in the sample tested). The treatment of *H. pylori* cells with sublethal concentrations of 3-BP conditioned the presence of a higher average number of spiral forms than in MICs, i.e., 58.75% and 77.5% (*H. pylori* Tx30a) and 55.25% and 75% (*H. pylori* 7143) for ½× MIC and ¼× MIC, respectively (Figure 8, Figure 9, Figure 10 and Figure 11). Among the tested antibiotics, the strongest inducer of *H. pylori* morphological changes was amoxicillin (at ½× MIC, 26.75% and 41.25% of spiral forms were observed for *H. pylori* Tx30a and 7143 strain, respectively), while the weakest inducers were MTZ relative to *H. pylori* Tx30a (74.25% and ≥90% spiral forms for ½× MIC and ¼× MIC, respectively) and CLR relative to *H. pylori* 7143 (78.5% and ≥90% spiral forms for ½× MIC and ¼× MIC, respectively) (Figure 8, Figure 9, Figure 10 and Figure 11). A similar mechanism of the coccoid form stimulation was noticed with the MICs of the 3-BP and each of the tested antibiotics combination. Lowering the concentration of one or both substances contributed to the gradual, inversely proportional to the substance concentration increase in the amount of *H. pylori* spiral forms (Figure 8, Figure 9, Figure 10 and Figure 11).

## 3. Discussion

*H. pylori* is an example of a microorganism that relatively often becomes resistant to antibiotics, which is most often obtained through point mutations in the target sites of antibiotics [27,28]. There is a strong dependence between the primary resistance of this bacterium and the level of consumption of specific antibiotics groups in the studied populations [29]. Primary resistance to CLR is associated with the use of this antimicrobial compound during lower respiratory tract infections, while primary resistance to MTZ is most often determined by the use of this antibiotic in urogenital and dental infections [28]. The secondary resistance to antibiotics is generated by the reinfection of *H. pylori* in people who have already had eradication therapy directed against this bacterium [30]. In the meta-analysis carried out by Savoldi et al. [13], it was estimated that the risk of therapeutic failure, regardless of the type of *H. pylori* resistance, increases 7-fold and 2.5-fold for the CLR-resistant and MTZ-resistant strains, respectively. This observation confirms the validity of searching for new antimicrobial substances with activity directed against the CLR-resistant *H. pylori* strains [14].

The present article determines the activity of 3-BP against antibiotic-susceptible and antibiotic-resistant *H. pylori* strains. It has been noticed that the resistance of this bacterium to antibiotics does not translate into the efficacy of this compound. This is consistent with the observations of other researchers, pointing to the lack of dependence between the resistance profile of this bacterium and the antimicrobial activity of nonantibiotic substances [31,32,33,34]. In *H. pylori* strains, the MICs of 3-BP were found to be in the range of 32–128 μg/mL, while in all tested strains, the MBCs counted for 128 μg/mL. The obtained MICs are slightly higher while in the similar range (20–80 μg/mL) as in *Staphylococcus aureus* (*S. aureus*) strains, against which the 3-BP activity was tested [26]. The authors of this article suggested the selective 3-BP activity directed against staphylococci because in the remaining tested bacteria (*Enterococcus, Enterobacter, Pseudomonas aeruginosa, Klebsiella pneumoniae*, and *Acinetobacter baumannii*), MICs were higher than 320 μg/mL [26]. The results in our article, therefore, indicate additional, selective activity against *H. pylori*. Furthermore, the MBC values of 3-BP against *H. pylori* strains, being 128 μg/mL (~0.77 mM), are achievable in vivo without inducing a toxic effect on eukaryotic cells because the threshold concentration was considered as 1.75 mM [35,36]. The study of Kunjithapatham et al. has shown that 3-BP has the ability to interact with serum proteins, which, according to the authors, is most likely responsible for the lack of cytotoxicity when the compound is administered systemically at a dose of ≤1.75 mM. Additionally, it was found that 3-BP does not cross the blood–brain barrier, which limits its neurotoxic potential [36]. It is believed that the reason for the high selectivity of 3-BP in the destruction of cancer cells is caused by the high expression of monocarboxylic acid transporters (MCTs), which the function of is to transport lactate molecules. Due to structural homology, 3-BP can also be transported by them. In physiologically functioning cells, the level of MCTs expression is low, which reduces the level of 3-BP transport to their interior and thus decreases the toxicity of 3-BP relative to them [16]. However, more research is still needed to finally determine safe dosages for the use of 3-BP in therapies.

During the tests determining MICs and MBCs of 3-BP against *H. pylori* strains, the MBC/MIC ratios ≤4 were obtained, indicating the bactericidal effect of this compound [37]. The time-killing assay confirmed the results and demonstrated the concentration-dependent and time-dependent activity of 3-BP against *H. pylori* strains. This antimicrobial mechanism of 3-BP activity has also been demonstrated against protozoa [20,21], fungi [22,23], and microalgae [24], as well as against clinical and laboratory *S. aureus* strains [26]. Microscopic observations of *H. pylori* treated with 3-BP indicated a decrease in the number of spiral forms with an inversely proportional increase in the amount of coccoid forms during the incubation. Morphological variability in response to unfavorable environmental conditions is typical for many Gram-negative rods, including *H. pylori* [38]. This microorganism, in response to stressful conditions, most often undergoes a morphological transformation into spherical forms, for which increased survivability and participation in the failure of antimicrobial therapies are suggested [39,40,41]. In many studies defining the antibacterial activity of substances, the mechanism of *H. pylori* morphological conversion from the spiral to coccoid form was noticed [42,43,44]. Therefore, it seems that an important stage of research seeking new, alternative antibacterial substances is the determination of viability, not culturability, of this bacterium [45]. In this current study, during the time-killing assay, the viability of *H. pylori* strains was determined using fluorescence microscopy. It was observed that after the one-day incubation of *H. pylori* with 3-BP, these bacteria have a low, reduced mean green/red fluorescence ratio, suggesting the bactericidal activity of this compound is independent from the induction of spherical forms. Although the mechanism of bactericidal activity of 3-BP has not been recognized, it is suggested that in microbial cells, similar changes as in cancer cells treated with this compound may occur [23,24,26]. The induction of metabolic catastrophe depending on interference with enzyme activity, the limitation of intracellular ATP, and the generation of free oxygen radicals may be determinants of the strong 3-BP antimicrobial activity [15,16].

The final stage of this present study was to determine the interaction between 3-BP and the most commonly used antibiotics in therapies directed against *H. pylori*, i.e., AMX, CLR, TET, and MTZ. A synergistic/additive interaction has been demonstrated with three tested antibiotics (AMX, CLR, and TET). These observations coincide with the results obtained by other researchers [24,26]. The checkerboard assay showed the existence of a synergistic/additive interaction of 3-BP with amphotericin B against the microalgae [24] and with ampicillin against *S. aureus* [26]. Similarly, the current study demonstrated this type of relationship between 3-BP and AMX. It seems that the reason for this phenomenon is the ability of amphotericin B [46] and aminopenicillins [47] to interfere with the integrity of the cellular structures and a disintegration-dependent increase in uptake of 3-BP into microbial cells. An analogous mechanism of sensitizing bacterial cells to antibiotics has been proven against methicillin-resistant *S. aureus* exposed to electroporation [48]. In the Visca et al. [26] study, the existence of an additive interaction of 3-BP with TET and chloramphenicol against *S. aureus* has also been demonstrated. These results confirm again the observations made in this present study, including the additive interaction of 3-BP with TET and CLR, an antibiotic acting similarly to chloramphenicol on the 50s ribosomal subunit. Inhibitors of protein synthesis, such as the aforementioned antibiotics, can affect the production of key proteins responsible for the defense reactions, including stress proteins [49]. It seems that this type of activity may sensitize bacteria to the bactericidal action of 3-BP and inhibit mechanisms aimed at eliminating the harmful effects that occur after exposure to this compound. This hypothesis, however, requires research verification in the future.

## 4. Materials and Methods

### 4.1. Bacterial Strains and Culture Conditions

The study was conducted using 52 *H. pylori* strains (50 clinical strains, isolated during previous studies [50,51], and two reference strains, Tx30a (ATCC 51932) and J99 (ATCC 700824)) (Table 1). The strains were categorized as susceptible or resistant to antibiotics based on the EUCAST recommendations, i.e., amoxicillin (AMX, R > 0.125 μg/mL), clarithromycin (CLR, R > 0.5 μg/mL), tetracycline (TET, R > 1 μg/mL), and metronidazole (MTZ, > 8 μg/mL) [52]. Bacterial strains were kept in a Trypticase soy broth (TSB) (Oxoid, Le Pont de Claix, France) with the addition of 15% glycerol at −70 °C until testing [41]. After thawing, the bacteria were plated on Columbia agar (Difco, Lublin, Poland) with 7% hemolysed horse blood (CA+HB) and incubated for 3 days under microaerophilic conditions (Genbox microaer kits, BioMerieux, Marcy I’Etoile, France) at 37 °C. The grown bacteria were passaged on CA+HB and again incubated in the aforementioned conditions for the next 3 days [41].

### 4.2. Disk-Diffusion Method

The activity of 3-BP (Sigma-Aldrich, St. Louis, MO, USA) using the disk-diffusion method was determined against 52 *H. pylori* strains. A suspension of the bacterial strain tested with an optical density equivalent to 4 McFarland standard (~10^8^ CFU/mL) was prepared in Brain heart infusion (BHI) broth (Oxoid) with 7% foetal calf serum (Gibco, Paisley, Scotland) (BHI+FCS) and swabbed on the surface of a freshly prepared CA+HB agar, thus obtaining a final bacterial density of approx. 5 × 10^6^ CFU/mL [53,54] with minor modifications. Three sterile paper discs (6 mm) were placed evenly on the agar surface, and 20 μL of different 3-BP solutions, corresponding to concentrations of 100 mg/mL (2000 μg/disk), 50 mg/mL (1000 μg/disk), and 10 mg/mL (200 μg/disk), were dropped on them. 3-BP was dissolved in DMSO (Sigma-Aldrich) and diluted to the final concentration not exceeding 1% (*v/v*). The positive and negative controls of the experiment were discs with AMX (Oxoid, 25 μg/disk) and 1% DMSO, respectively. All culture plates with sown bacteria were incubated for 3 days under microaerophilic conditions at 37 °C, and then the growth inhibition zones were measured. The experiment was carried out in duplicate.

### 4.3. MIC/MBC Determination

The minimal inhibitory concentrations (MICs) and minimal bactericidal concentrations (MBCs) were determined against 10 selected *H. pylori* strains (two reference strains, Tx30a and J99, and 8 clinical isolates: two susceptible to antibiotics, two MTZ-resistant, two CLR-resistant, and two with resistance to both antibiotics) (Table 2). The study was carried out using the microdilution method in 12-well titration plates (Bionovo, Legnica, Poland) [55]. For each *H. pylori* strain, a suspension with an optical density of 4 McFarland units (approx. 10^8^ CFU/mL) in BHI+FCS broth was prepared and then 0.1 mL of the bacterial suspension was transferred to each well with 0.9 mL of BHI+FCS and a 3-BP concentration gradient (8–512 μg/mL), thereby obtaining 1 mL of a bacterial suspension with a final density of approx. 10^7^ CFU/mL. The microdilution plates were incubated for 3 days under microaerophilic conditions at 37 °C with shaking (100 rpm). The positive control was BHI+FCS broth alone and BHI+FCS broth with 1% DMSO (*v/v*), both with tested bacteria, while the negative control was BHI+FCS without bacteria. The experiment was carried out in duplicate. The MIC was traced as the lowest concentration in which no bacterial growth was observed [56]. To determine the MBC, 10 μL from each well of the microtiter plate was dropped on CA+HB agar and incubated for 3 days at 37 °C and microaerophilic conditions. The MBC was considered as the lowest concentration in which no bacterial growth was observed on the agar plate [56].

The determination of the 3-BP activity in an environment with different pH values was performed using two reference *H. pylori* strains (Tx30a and J99). For this purpose, BHI+FCS broths with different pH values (5, 6, 7, and 8) were prepared, which was obtained with 1 M HCl and NaOH solutions. The culture conditions, culture media, and bacterial optical density were identical to those used in the MIC/MBC determination. The experiment was carried out in duplicate.

### 4.4. Checkerboard Assay

The existence of synergism in the antimicrobial activity of 3-BP with AMX (Sigma-Aldrich), MTZ (Sigma-Aldrich), TET (Sigma-Aldrich), and CLR (Sigma-Aldrich) was determined against two *H. pylori* strains (antibiotic-susceptible reference strain (Tx30a) and double-resistant clinical strain (7143)) using the checkerboard assay [57,58,59]. A concentration gradient used for the 3-BP was similar as previously (i.e., 8–512 μg/mL). The ranges of antibiotic concentrations were chosen based on the study by Hirschl et al. [57], with modifications made during experimental studies, and they counted for 0.00188–0.12 μg/mL, 0.00625–0.4 μg/mL, 0.00313–0.2 μg/mL, and 0.125–8 μg/mL for the Tx30a strain and 0.00313–0.2 μg/mL, 0.025–1.6 μg/mL, 8–512 μg/mL, and 8–512 μg/mL for the 7143 strain for AMX, TET, CLR, and MTZ, respectively.

The concentration gradients of the antimicrobial substances were prepared in test tubes in a way to obtain twice as high concentrations when testing the activity of 3-BP or selected antibiotics alone and four times higher concentrations when testing the combination of 3-BP and a chosen antibiotic. The experiment was carried out using six 12-well titration plates forming a total of 72-well panels. The external wells of the 12-well plates of the x- and y-axes had a concentration gradient of 3-BP and the antibiotic, respectively. To each of these wells, 0.5 mL of BHI+FCS with the tested, doubled 3-BP or antibiotic concentration, 0.4 mL of BHI+FCS, and 0.1 mL of BHI+FCS with a 4 McFarland bacterial suspension were added, thus obtaining the desired concentrations of antimicrobials and final bacterial density of approx. 10^7^ CFU/mL. To the remaining wells, in which the interactions in the antimicrobial activity were determined, 0.25 mL of BHI+FCS with a tested, four-fold 3-BP concentration, 0.25 mL of BHI+FCS with a tested four-fold antibiotic concentration, 0.4 mL of BHI+FCS, and 0.1 mL of BHI+FCS with a 4 McFarland bacterial suspension were added, thus obtaining the desired concentrations of antimicrobial substances and final bacterial density of approx. 10^7^ CFU/mL. The plates were incubated for 3 days at 37 °C in microaerophilic conditions. Each tested panel was performed in duplicate.

On the basis of the obtained results, the MICs for 3-BP, antibiotic, and the combination of both substances were determined. The interaction between the tested antimicrobial agents was determined by calculating the FIC index (MIC of substance A in combination/MIC of substance A alone + MIC of substance B in combination/MIC of substance B alone). The values of the FIC index were interpreted as ≤0.5 = synergistic, ˃0.5 to ≤1 = additive, ˃1 to ˂4 = neutral, and ≥4 = antagonistic [57,58,59].

### 4.5. Time-Killing Assay

The determination of the culturability during exposure to 3-BP over time was performed against two reference *H. pylori* strains (Tx30a and J99) based on the study by Brown et al. [60] with minor modifications. The culture conditions, culture media, and bacterial optical density were identical to those used in the MIC/MBC determination, with the exception being the use of a 2 mL culture volume in each well of the 12-well titrate plate (instead of 1 mL). At each time point (0 h, 1 h, 2 h, 4 h, 6 h, 8 h, and 24 h), 0.1 mL of cultures without the 3-BP presence (control) and cultures from each tested 3-BP concentration, i.e., 4× MIC (512 μg/mL), 2× MIC (256 μg/mL), MIC (128 μg/mL), and ½× MIC (64 μg/mL), were taken and a set of culture dilutions in BHI+FCS broths were made. The 0.1 mL of appropriate dilutions (Control: 10^−3^, 10^−4^, and 10^−5^ for all time points. ½× MIC: 10^−3^, 10^−4^, and 10^−5^ (0 h); 10^−2^, 10^−3^, and 10^−4^ (1 h, 2 h, and 4 h); and 10^−1^, 10^−2^, and 10^−3^ (6 h, 8 h, and 24 h). MIC-4× MIC: 10^−3^, 10^−4^, and 10^−5^ (0 h); 10^−1^, 10^−2^, and 10^−3^ (1 h, 2 h, and 4 h); and 10^−1^ and 10^−2^ (6 h, 8 h, and 24 h)) were seeded on CA+HB agars and incubated for 3 days at 37 °C in microaerophilic conditions. The amount of grown *H. pylori* colonies was counted and presented as log_10_ CFU/mL. The experiment was carried out in duplicate.

### 4.6. Light Microscopy

In the checkerboard and time-killing assays, the bacterial morphology was determined based on the study by Krzyżek et al. [41] with minor modifications. The slides were covered with 50 μL of bacterial suspension from each tested antimicrobial concentration and stained using the Gram’s method. In the checkerboard assay, from each tested antimicrobial concentration, two preparations were made in each repetition and the morphology of 100 cells/preparation was determined (*n* = 400). In wells in which the number of spiral forms counted for ≤15% or ≥90%, the exact number of these morphological forms was not determined. In the time-killing assay, in each repetition, one preparation from each concentration and time point was made and the morphology of 100 cells/preparation was determined (*n* = 200). The exception was the 0 h time point, where one preparation was made from each concentration tested, determining the morphology of 100 cells/preparation and presenting this value as the average number of spiral forms at the beginning of the experiment (*n* = 500). The study was performed under the Olympus BX50 microscope (Olympus Optical, Tokyo, Japan), using an oil-immersion ×100 lens with a numerical aperture of 1.3.

### 4.7. Fluorescence Microscopy

To determine the viability of *H. pylori* strains during the time-killing assay, the study was extended by fluorescence analysis using BacLight Live/Dead staining kit (L7012, ThermoFisher, Waltham, MA, USA). This kit consists of two components: SYTO9 (green fluorescent dye), which stains all bacteria, and propidium iodide (PI, red fluorescent dye), staining only bacteria with damaged cell membranes. The test was carried out in accordance with the manufacturer’s instructions. Briefly, 0.1 mL of culture from each concentration and time point was taken and centrifuged for 15 min at 10,000 *g*. The supernatant was removed, and the resulting bacterial pellet was resuspended in 1 mL 0.85% NaCl solution and subjected to 10,000 *g* centrifugation for 15 min. This procedure was performed twice. Then, the supernatant was collected, and the bacterial pellet was resuspended in 0.2 mL 0.85% NaCl solution. The 0.6 μL mixture of propidium iodide and SYTO9 (1:1 ratio) was added to the bacterial suspension and incubated for 15 min in the dark. The positive control consisted of bacteria from 0 h incubation, while the negative control was obtained by an 1 h incubation of the bacterial pellet from 0 h incubation in 70% ethanol (Chempur, Piekary Śląskie, Poland). The preparations were made by dropping 10 μL of bacterial suspensions and covering with coverslips.

The microscopic examination was performed on the basis of studies by Chou et al. and Marchesini et al. [61,62] with modifications. The preparations were examined under the Olympus BX51 microscope (Olympus Optical, Tokyo, Japan) using a ×10 lens with a numerical aperture of 0.3. Using the ImageJ software, the intensity of the green and red fluorescence from each preparation was counted for 25 regions of interests (ROIs) (50 ROIs/tested sample) that included single bacterial cells or small bacterial aggregates. The fluorescence intensities of SYTO9 and propidium iodide were measured at an emission of 530 nm and 640 nm, respectively. The fluorescence intensity of the tested samples was presented as the mean of the green/red fluorescence ratio.

### 4.8. Scanning Electron Microscopy

The bacterial morphology using scanning electron microscopy was determined based on the study by Krzyżek et al. [41] with minor modifications. Centrifuged bacterial suspensions were fixed for 24 h in 2.5% glutaraldehyde in 0.1 M cacodylate buffer at physiological pH. The material was then dehydrated in the growing alcoholic series (10% > 30% > 50% > 70% > 90% > 99.8%). Samples were spotted on aluminum tables, dried, dusted with carbon (15 nm), and placed in the scanning chamber electron microscope (Auriga 60, Zeiss, Oberkochen, Germany). The analysis of the bacterial morphology was carried out at the beam voltage equal to 2 kV and the working distance of 5 mm.

### 4.9. Statistical Analysis

The Kruskal–Wallis test and the Mann–Whitney *U* test were used in the statistical analysis of the differences in the 3-BP activity between *H. pylori* strains during the disk-diffusion method and broth microdilution method, respectively. The effect of 3-BP on the culturability of *H. pylori* was analyzed by the Kaplan–Meier method and the Wilcoxon test. The statistical significance of a categorical data was assessed using the chi-square Pearson test. The significance level was set to be 5%.

## 5. Conclusions

To the authors’ knowledge, this study is the first survey focusing on the activity of 3-BP (alone or in the combination with antibiotics) against *H. pylori* strains. The results shown in the present in vitro study indicate a high bactericidal activity of 3-BP against antibiotic-susceptible and antibiotic-resistant *H. pylori* strains. In addition, a synergistic/additive interaction of this compound with AMX, TET, and CLR has been demonstrated. These observations indicate the potential for using 3-BP as a promising antimicrobial agent in therapies directed against *H. pylori*. For this reason, future research should focus on determining the in vivo activity of this substance.

## Figures and Tables

**Table 1:** The zones of inhibition (mm) of 3-Bromopyruvate (3-BP) against clinical and control *H. pylori* strains.

Strains	Antibiotic Resistance *		3-BP									AMX **		
			200 µg/disk			1000 µg/disk			2000 µg/disk			25 µg/disk		
	MTZ	CLR	Sample		Average	Sample		Average	Sample		Average	Sample		Average
			I	II		I	II		I	II		I	II	
1950	R	R	6	6	6	17	21	19	21	24	22,5	69	70	69.5
1952	S	R	11	13	12	20	23	21.5	23	21	22	67	71	69
1954	R	R	13	12	12.5	13	13	13	18	22	20	68	67	67.5
1964	R	R	6	6	6	14	15	14.5	24	20	22	70	71	70.5
2093	R	S	8	10	9	11	12	11.5	16	16	16	62	64	63
2095	S	S	11	10	10.5	18	15	16.5	25	28	26.5	70	69	69.5
6010	R	S	9	10	9.5	14	15	14.5	18	21	19.5	66	65	65.5
6171	S	S	10	10	10	14	14	14	18	17	17.5	65	65	65
6237	S	S	9	10	9.5	18	17	17.5	26	28	27	68	72	70
6291	R	R	16	15	15.5	23	25	24	26	27	26.5	69	70	69.5
6341	R	R	9	9	9	25	21	23	34	31	32.5	71	70	70.5
6343	S	S	7	9	8	19	21	20	29	32	30.5	69	71	70
6522	R	R	7	8	7.5	15	16	15.5	20	18	19	65	64	64.5
6559	R	S	8	8	8	17	16	16.5	28	31	29.5	66	69	67.5
6574	S	S	8	8	8	14	16	15	17	19	18	60	64	62
6575	S	S	8	9	8.5	15	15	15	29	33	31	64	63	63.5
6638	S	R	8	8	8	16	15	15.5	27	30	28.5	70	69	69.5
6649	S	S	6	7	6.5	13	14	13.5	18	18	18	66	66	66
6653	S	R	7	7	7	13	13	13	25	29	27	68	70	69
6687	R	R	7	8	7.5	15	16	15.5	20	23	21.5	68	69	68.5
6699	S	R	7	9	8	18	20	19	22	25	23.5	69	70	69.5
6716	S	S	18	20	19	29	28	28.5	30	32	31	71	70	70.5
6735	S	S	10	10	10	15	15	15	22	24	23	67	68	67.5
6741	S	S	9	10	9.5	13	14	13.5	19	19	19	63	65	64
6794	R	R	9	8	8.5	12	12	12	17	19	18	63	66	64.5
6858	S	R	7	7	7	13	14	13.5	17	17	17	61	58	59.5
6875	R	S	9	10	9.5	12	12	12	18	19	18.5	68	72	70
6885	S	S	6	6	6	10	10	10	16	17	16.5	69	72	70.5
7042	S	S	11	9	10	13	14	13.5	18	17	17.5	61	64	62.5
7080	R	S	10	12	11	15	13	14	16	17	16.5	62	62	62
7101	S	S	9	10	9.5	13	14	13.5	18	18	18	65	64	64.5
7110	S	S	11	11	11	14	16	15	22	23	22.5	67	68	67.5
7143	R	R	8	8	8	13	13	13	18	17	17.5	66	67	66.5
7173	R	S	9	11	10	13	14	13.5	17	17	17	60	62	61
7189	S	R	11	10	10.5	12	12	12	20	18	19	64	63	63.5
7208	S	S	8	9	8.5	14	15	14.5	21	24	22.5	67	63	65
7264	S	S	9	9	9	11	13	12	17	18	17.5	69	70	69.5
7286	R	S	8	9	8.5	14	14	14	19	17	18	69	68	68.5
7297	S	S	10	11	10.5	17	17	17	20	22	21	63	61	62
7308	S	S	9	9	9	12	12	12	18	19	18.5	57	59	58
7317	S	R	11	12	11.5	15	14	14.5	19	19	19	59	60	59.5
7357	R	S	11	10	10.5	16	17	16.5	19	20	19.5	69	70	69.5
7361	R	S	9	9	9	15	15	15	19	18	18.5	69	71	70
7388	R	S	8	8	8	13	14	13.5	17	17	17	71	70	70.5
7394	R	S	14	13	13.5	26	24	25	29	33	31	67	64	65.5
7404	S	S	11	10	10.5	17	17	17	24	27	25.5	68	67	67.5
7471	S	S	12	13	12.5	21	22	21.5	27	30	28.5	57	60	58.5
7556	S	R	17	15	16	24	23	23.5	29	27	28	71	70	70.5
7649	R	R	10	10	10	16	16	16	25	26	25.5	64	61	62.5
8064	R	R	7	8	7.5	16	15	15.5	20	22	21	60	62	61
J99	S	S	6	6	6	11	11	11	19	18	18.5	65	68	66.5
Tx30a	S	S	10	12	11	14	15	14.5	24	23	23.5	68	71	69.5

* The antibiotic resistance: S is susceptible, R is resistant, MTZ is Metronidazole, and CLR is Clarithromycin. ** Amoxicillin (AMX) was a positive control of the study. The negative control was a 1% DMSO solution (*v/v*) that did not cause the appearance of the growth inhibition zone in all tested *H. pylori* strains (6 mm).

**Table 2:** The minimal inhibitory concentrations (MICs) and minimal bactericidal concentrations (MBCs) of 3-Bromopyruvate (3-BP) against selected *H. pylori* strains.

Strains	Antibiotic Resistance *		Activity of 3-BP		
	MTZ	CLR	MIC**	MBC **	MBC/MIC Ratio
J99	S	S	128	128	1
Tx30a	S	S	128	128	1
6237	S	S	32	128	4
7471	S	S	64	128	2
7189	S	R	32	128	4
7556	S	R	128	128	1
7388	R	S	128	128	1
7394	R	S	64	128	2
7143	R	R	128	128	1
7649	R	R	128	128	1

* The antibiotic resistance: S is susceptible, R is resistant, MTZ is Metronidazole, and CLR is Clarithromycin. ** The MIC and MBC concentrations are given in µg/mL.

## Supplementary Materials

The following are available online at http://www.mdpi.com/2072-6694/11/2/229/s1: Table S1: The zones of inhibition (mm) of 3-Bromopyruvate (3-BP) against antibiotic-susceptible *H. pylori* strains, Table S2: The zones of inhibition (mm) of 3-Bromopyruvate (3-BP) against metronidazole-resistant *H. pylori* strains, Table S3: The zones of inhibition (mm) of 3-Bromopyruvate (3-BP) against clarithromycin-resistant *H. pylori* strains, Table S4: The zones of inhibition (mm) of 3-Bromopyruvate (3-BP) against CLR- and MTZ-resistant *H. pylori* strains, Table S5: The effect of 3-Bromopyruvate (3-BP) on the culturability of *H. pylori* Tx30a, Table S6: The effect of 3-Bromopyruvate (3-BP) on the culturability of *H. pylori* J99, Table S7: The effect of 3-Bromopyruvate (3-BP) on the number of spiral forms of *H. pylori* Tx30a, Table S8: The effect of 3-Bromopyruvate (3-BP) on the number of spiral forms of *H. pylori* J99, Table S9: The analysis of the mean green/red fluorescence ratios during the incubation of *H. pylori* Tx30a with 3-Bromopyruvate (3-BP) in time, Table S10: The analysis of the mean green/red fluorescence ratios during the incubation of *H. pylori* J99 with 3-Bromopyruvate (3-BP) in time, Table S11: The morphology of *H. pylori* T30a after a 3-day incubation with 3-BP and CLR during the checkerboard assay, Table S12: The morphology of *H. pylori* T30a after a 3-day incubation with 3-BP and TET during the checkerboard assay, Table S13: The morphology of *H. pylori* T30a after a 3-day incubation with 3-BP and AMX during the checkerboard assay, Table S14: The morphology of *H. pylori* T30a after a 3-day incubation with 3-BP and MTZ during the checkerboard assay, Table S15: The morphology of *H. pylori* 7143 after a 3-day incubation with 3-BP and CLR during the checkerboard assay, Table S16: The morphology of *H. pylori* 7143 after a 3-day incubation with 3-BP and TET during the checkerboard assay, Table S17: The morphology of *H. pylori* 7143 after a 3-day incubation with 3-BP and AMX during the checkerboard assay, and Table S18: The morphology of *H. pylori* 7143 after a 3-day incubation with 3-BP and MTZ during the checkerboard assay.
